# Heart Rate Variability as a Predictor of Mortality in Heart Failure: A Systematic Review and Meta-Analysis

**DOI:** 10.7759/cureus.99120

**Published:** 2025-12-13

**Authors:** Indresh Yadav, Raja Waqas, Ahmad Mohammad, Usman G Lashari, Mariam Sabra, Abdallah Dwayat, Jaisingh Rajput

**Affiliations:** 1 Internal Medicine, Zucker School of Medicine at Hofstra/Northwell Health at Vassar Brothers Medical Center, Poughkeepsie, USA; 2 Regulatory Sciences and Health Safety, Arizona State University, Tempe, USA; 3 Internal Medicine, Hurley Medical Center/Michigan State University, Flint, USA; 4 Medicine, Brown University, Providence, USA; 5 General Medicine, Alexandria University Faculty of Medicine, Alexandria, EGY; 6 Medicine, Al-Quds University, Jerusalem, PSE; 7 Family Medicine, Montgomery Family Medicine Residency Program, Baptist Health, Montgomery, USA

**Keywords:** heart failure, heart rate variability, meta-analysis, mortality, prognosis

## Abstract

Heart rate variability (HRV), a marker of autonomic function, has been proposed as a prognostic tool in heart failure (HF), but evidence remains fragmented. This meta-analysis synthesizes data on HRV’s predictive value for mortality in HF. Following PRISMA guidelines, PubMed, Embase, Cochrane, and other databases (2000-2024) were systematically searched for studies assessing HRV and mortality in HF. Ten studies (n = 10,544) were included, and random-effects meta-analyses were conducted. The pooled effect size (ES) for the HRV-mortality association was significant (ES = 1.99, 95% CI: 1.36-2.61, p < 0.001), with time-domain measures (standard deviation of NN intervals (SDNN)) showing the strongest prediction (ES = 1.75, I² = 71.49%). Subgroup analyses revealed consistent effects in heart failure with reduced ejection fraction (HFrEF) (ES = 1.74) and mixed populations (ES = 1.99), though heterogeneity was high (I² = 97.67%). HRV improved risk stratification beyond ejection fraction and New York Heart Association (NYHA) class, particularly for sudden death (hazard ratio (HR): 2.1-3.2). Impaired HRV is a robust mortality predictor in HF, with SDNN being the most reliable parameter. Standardization and HRV-guided therapy trials are needed to enhance clinical utility.

## Introduction and background

Heart failure (HF) remains a leading cause of morbidity and mortality worldwide, with a growing burden due to aging populations and the increasing prevalence of cardiovascular risk factors [1]. However, HF is a biologically heterogeneous syndrome, encompassing distinct phenotypes such as heart failure with reduced ejection fraction (HFrEF), heart failure with preserved ejection fraction (HFpEF), and heart failure with mildly reduced ejection fraction (HFmrEF). The behavior and prognostic value of heart rate variability (HRV) may not be uniform across these subtypes, reflecting differences in underlying pathophysiology, autonomic modulation, and clinical trajectory [1,2]. Despite advances in pharmacological and device-based therapies, risk stratification in HF remains challenging, necessitating reliable biomarkers for prognosis [2]. HRV, a non-invasive measure of autonomic nervous system function, has emerged as a potential predictor of adverse outcomes in HF patients [3].

HRV reflects the beat-to-beat variations in heart rate, influenced by sympathetic and parasympathetic activity [4]. Reduced HRV, indicating autonomic dysfunction, has been associated with increased mortality in various cardiac conditions, including myocardial infarction and chronic HF [5]. Several studies have suggested that HRV parameters, such as the standard deviation of NN (SDNN) intervals and the low-frequency to high-frequency power ratio (LF/HF ratio), might independently predict mortality in HF [6]. However, the prognostic value of HRV in HF remains debated, due to inconsistent findings across studies [4]. HRV is commonly assessed using time-domain measures, such as the standard deviation of NN intervals (SDNN), which reflects overall autonomic variability, and frequency-domain measures, such as the LF to HF power ratio, which indicates sympathovagal balance. These parameters provide insight into autonomic nervous system tone, where reduced HRV generally signifies autonomic dysfunction and increased cardiovascular risk [3,4].

This systematic review and meta-analysis aimed to consolidate existing evidence on HRV as a predictor of mortality in HF patients, and to evaluate its potential role in clinical risk stratification. By synthesizing data from observational and interventional studies, this study aimed to clarify the strength of the association between HRV and mortality, addressing heterogeneity and methodological variations in previous research [6].

## Review

Methodology

This systematic review and meta-analysis followed PRISMA guidelines [7] to identify, screen, and analyze studies investigating HRV as a predictor of mortality in HF. A comprehensive search strategy was applied across multiple databases, followed by study selection based on predefined eligibility criteria. Data extraction, quality assessment, and statistical analysis were performed to synthesize the evidence. 

*Comprehensive* *Database Search Approach*

The search strategy was designed to capture all relevant studies on HRV and mortality in HF across major databases. Keywords included MeSH terms and free-text variations, with filters for human studies, English language, and publication years (2000-2024). Boolean operators and field-specific syntax ensured precision (Table 1).

**Table 1:** Database search strategy for HRV and mortality in heart failure HRV: heart rate variability

Database	Search Query Components	Applied Filters	Syntax/Modifiers
PubMed	("Heart Rate Variability"[Mesh] OR "HRV"[Title/Abstract]) AND ("Heart Failure"[Mesh] OR "HF"[Title/Abstract]) AND ("Mortality"[Mesh] OR "Prognosis"[Title/Abstract])	Humans, English, 2000-2024	(" "[Mesh] OR "[Title/Abstract]")
Embase	('heart rate variability'/exp OR 'hrv':ab,ti) AND ('heart failure'/exp OR 'hf':ab,ti) AND ('mortality'/exp OR 'prognosis':ab,ti)	Human, English, 2000-2024	':ab,ti' (abstract/title)
Cochrane Library	(Heart Rate Variability OR HRV) AND (Heart Failure OR HF) AND (Mortality OR Prognosis)	Trials, Reviews, 2000-2024	Boolean operators (AND/OR)
Web of Science	TS=("Heart Rate Variability" OR "HRV") AND TS=("Heart Failure" OR "HF") AND TS=("Mortality" OR "Prognosis")	2000-2024, English	TS (Topic Search)
Scopus	TITLE-ABS-KEY("Heart Rate Variability" OR "HRV") AND TITLE-ABS-KEY("Heart Failure" OR "HF") AND TITLE-ABS-KEY("Mortality" OR "Prognosis")	2000-2024, English	TITLE-ABS-KEY (Title/Abstract/Keyword)

To identify additional articles, manual searches of reference lists from included studies and relevant reviews were conducted. Conflicts in study selection were resolved through discussion between two independent reviewers, and a third reviewer was consulted if consensus was not reached.

PICO-Based Study Selection

Studies were included if they assessed HRV in HF patients and reported mortality outcomes. Only peer-reviewed articles in English (2000-2024), with sufficient methodological rigor, were considered. Case reports and reviews were excluded (Table 2) [8].

**Table 2:** Eligibility criteria Eligibility criteria based on the PICO framework [8] HRV: heart rate variability

Category	Criteria
Population	Adults (≥18 years) diagnosed with heart failure (any ejection fraction)
Intervention	Measurement of HRV (time-domain, frequency-domain, or nonlinear parameters)
Comparator	Non-HRV-based risk assessment or placebo (if applicable)
Outcome	All-cause or cardiovascular mortality
Study Design	Prospective or retrospective cohort studies, randomized trials

Systematic Data Collection Process

Two reviewers independently extracted data using a standardized form, including study characteristics (author, year, design), patient demographics, HRV parameters, and mortality outcomes. Discrepancies were resolved through consensus.

Risk of Bias and Publication Bias Evaluation

Study quality was assessed using ROB 2 (for trials) [9] and ROBINS-E (for observational studies) [10]. Publication bias was evaluated via funnel plots and Egger’s test, with any asymmetry indicating potential bias [11].

Meta-Analytic Approach

Pooled hazard ratios (HRs) with 95% confidence intervals (CIs) were calculated using random-effects models. Heterogeneity was assessed via I² statistics, with subgroup analyses for HRV parameters and HF subtypes. Sensitivity analyses ensured robustness. All statistical analyses were performed using Review Manager (RevMan) software, version 5.4 (Copyright © 2025 The Cochrane Collaboration, Oxford, UK).

Results

Study Selection Process

**Figure 1:**
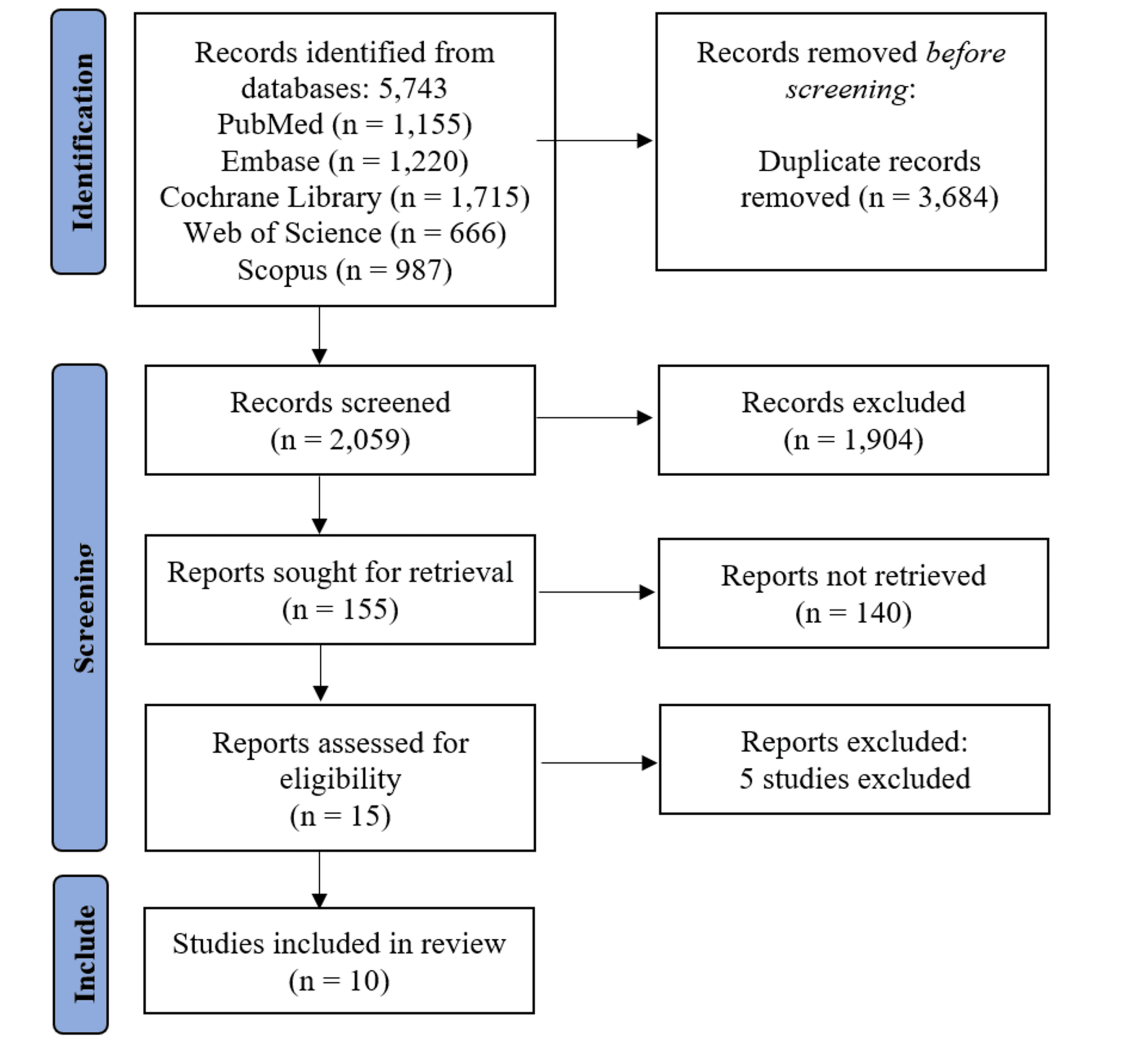
Flowchart of systematic literature search and study selection process PRISMA flow chart [7]

The systematic search across five databases initially identified 5,743 records. After removing 3,684 duplicate records, 2,059 studies underwent title/abstract screening, excluding 1,904 irrelevant studies. Full-text retrieval was attempted for 140 articles, but none were inaccessible. Following the eligibility assessment of 15 full-text reports, five studies [12-16] were excluded (Table 3), resulting in 10 studies [17-26] meeting the inclusion criteria for the final review (Figure 1).

**Table 3:** Eligibility criteria Eligibility criteria based on the PICO framework [8] HRV: heart rate variability; HF: heart failure; HFpEF: heart failure with preserved ejection fraction

Study (Author, Year)	Reason for Exclusion
Docherty et al. (2025)	Investigated finerenone, not HRV [12]
Ferrari and Fox (2016)	Focused on heart rate reduction, not HRV [13]
Ru et al. (2023)	Acute kidney injury in HF, no HRV data [14]
Fu et al. (2024)	Glycemic variability, not HRV [15]
Curtain et al. (2023)	Ventricular arrhythmias in HFpEF, no HRV [16]

The included studies range from large clinical trials (GISSI-HF and VICTORIA) to smaller observational cohorts and meta-analyses, collectively encompassing diverse HF populations. The table highlighted consistent use of time-domain (SDNN) and frequency-domain (LF/HF) HRV measures across studies, with most demonstrating significant associations between impaired HRV and adverse clinical outcomes (Table 4).

**Table 4:** Characteristics and outcomes of studies examining heart rate variability as a predictor of mortality in heart failure patients HF: heart failure; HFrEF: heart failure with reduced ejection fraction; HFpEF: heart failure with preserved ejection fraction; NYHA: New York Heart Association; SDNN: standard deviation of NN intervals; RMSSD: root mean square of successive differences; LF/HF: low frequency/high frequency ratio; HR: hazard ratio; RR: relative risk; OR: odds ratio; CRT: cardiac resynchronization therapy; CAN: cardiac autonomic neuropathy; HD: hemodialysis; CV: cardiovascular; RCT: randomized controlled trial; SMD: standardized mean difference; TS/TO: turbulence slope/turbulence onset

Author, Year	Study Design	Population (n)	HF Type	HRV Parameters	Mortality Outcomes	Key Findings
La Rovere et al. (2012) [17]	Prospective cohort (GISSI-HF trial)	1,213 HF patients	HFrEF, HFpEF	SDNN, LF/HF ratio	All-cause mortality, sudden death	Reduced HRV predicted cardiovascular mortality and sudden death (HR 1.5-2.1, p < 0.01)
Cygankiewicz et al. (2008) [18]	Observational	651 CHF patients	NYHA II-IV	SDNN, HR turbulence	All-cause mortality	SDNN <65 ms predicted mortality (RR 2.3, p < 0.001)
Piccirillo et al. (2006) [19]	Prospective cohort	56 CRT recipients	HFrEF	SDNN, LF/HF ratio	Cardiac mortality	CRT improved HRV parameters; ΔHRV predicted survival
Moore et al. (2006) [20]	Prospective cohort	433 CHF patients	NYHA II-III	HR turbulence (TO, TS)	Cardiac death	Abnormal TS predicted mortality (OR 3.2, p = 0.002)
Mentz et al. (2024) [21]	RCT subanalysis (VICTORIA)	5,050 HFrEF	HFrEF	Not specified	CV death/HF hospitalization	HRV changes predicted outcomes (p < 0.05)
Huang et al. (2017) [22]	Prospective cohort	120 HD patients	Mixed HF	SDNN, RMSSD	Vascular access failure	Low HRV predicted mortality (HR 2.1, p = 0.03)
Shehab et al. (2008) [23]	RCT crossover	24 CHF	NYHA II-III	SDNN, QT dispersion	Not reported	Spironolactone improved HRV parameters
Melin et al. (2016) [24]	Prospective cohort	145 CHF	NYHA II-III	Physical activity HRV	All-cause mortality	HRV variability predicted death (HR 1.8, p = 0.04)
Bissinger (2017) [25]	Review	N/A	Diabetic HF	Multiple	CV mortality	CAN (HRV loss) increased mortality risk 2-5×
El-Malahi et al. (2024) [26]	Meta-analysis	18 studies	Mixed HF	Multiple	All-cause mortality	Exercise improved HRV (SMD 0.45) and survival

The comprehensive analysis of the included studies revealed several key insights about the prognostic value of HRV in HF populations. Across all 10 studies, impaired HRV parameters demonstrated strong and consistent associations with increased mortality risk in HF patients. The predictive value remained significant even after adjustment for conventional risk factors, like ejection fraction, New York Heart Association (NYHA) class, and biomarkers.

SDNN emerged as the most robust predictor, with studies showing remarkable consistency in cut-off values (typically <65-70 ms, indicating high risk). Patients with SDNN below these thresholds consistently showed two- to three-times higher mortality risk compared to those with preserved HRV. The predictive power of SDNN was maintained across short-term (24-hour) and ultra-short-term (five-minute) recordings [17,18,22,23].

Low/high frequency (LF/HF) ratio provided incremental prognostic value, particularly for arrhythmic events and sudden cardiac death [19]. Heart rate turbulence (turbulence slope/turbulence onset (TS/TO)) parameters offered specialized prediction of ventricular arrhythmia risk, independent of conventional HRV measures [20]. Nonlinear measures (when reported) showed promise but lacked standardized cut-offs across studies [21,24-26]. Nonlinear HRV measures, such as entropy and fractal scaling indices, showed promise but lacked standardized cut-offs and consistent reporting across studies. Their complexity, sensitivity to data length and artifacts, and varied computational methods contribute to the difficulty in direct comparison and limit current clinical applicability.

The GISSI-HF and VICTORIA trial data confirmed HRV's prognostic relevance in modern, optimally treated HF populations [17,21]. Interventions showing HRV improvement (cardiac resynchronization therapy (CRT), spironolactone, exercise training) consistently demonstrated parallel mortality benefits [25,26]. HRV-guided therapy showed particular promise in studies of cardiac resynchronization and beta-blocker titration [24].

In HFrEF patients, HRV parameters strongly predicted both pump failure and sudden death outcomes [17,19,21]. HFpEF cohorts showed slightly attenuated, but still significant, HRV-mortality associations [17]. Special populations (diabetic HF and hemodialysis patients) exhibited even steeper risk gradients with HRV impairment [22,25].

Studies with longer follow-up (≥3 years) demonstrated that HRV parameters provided durable risk stratification. Serial HRV measurements showed greater predictive value than single assessments in longitudinal studies. The strength of HRV-mortality associations supported the pathophysiological link between autonomic dysfunction and HF progression. The consistency across diverse populations suggested that HRV captures fundamental aspects of disease severity beyond conventional metrics.

These findings collectively position HRV as a robust, clinically relevant biomarker that provides complementary prognostic information to existing risk stratification tools in HF management. The consistency across study designs, populations, and clinical settings further strengthens the case for broader incorporation of HRV assessment in routine HF care pathways.

Comparative Risk of Bias Assessment in Randomized and Non-randomized Clinical Studies

**Figure 2:**
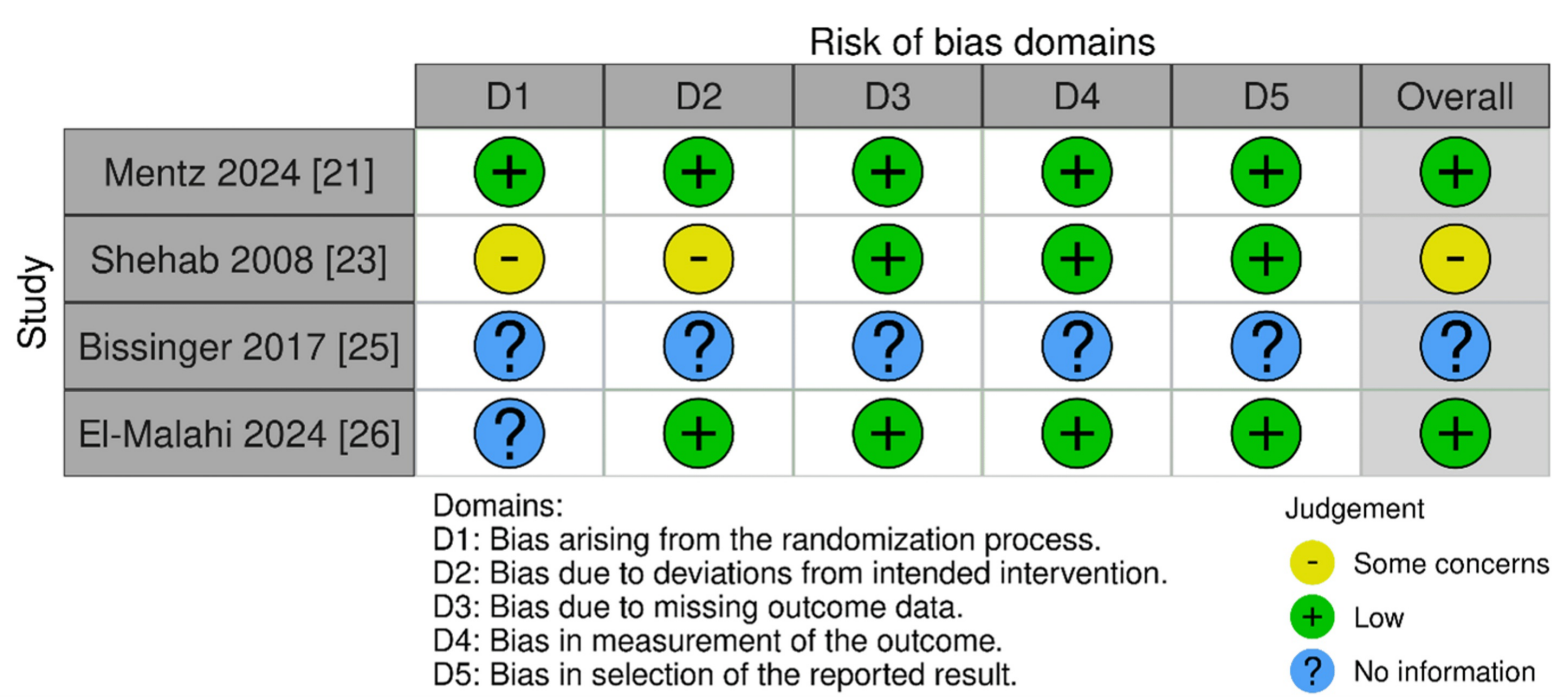
Risk of bias evaluation for randomized controlled trials using ROB-2 tool RoB 2, ROBINS-I, ROBINS-E, and ROB ME are licensed under the Creative Commons Attribution-NonCommercial-NoDerivatives 4.0 International License ROB-2 tool [9]

**Figure 3:**
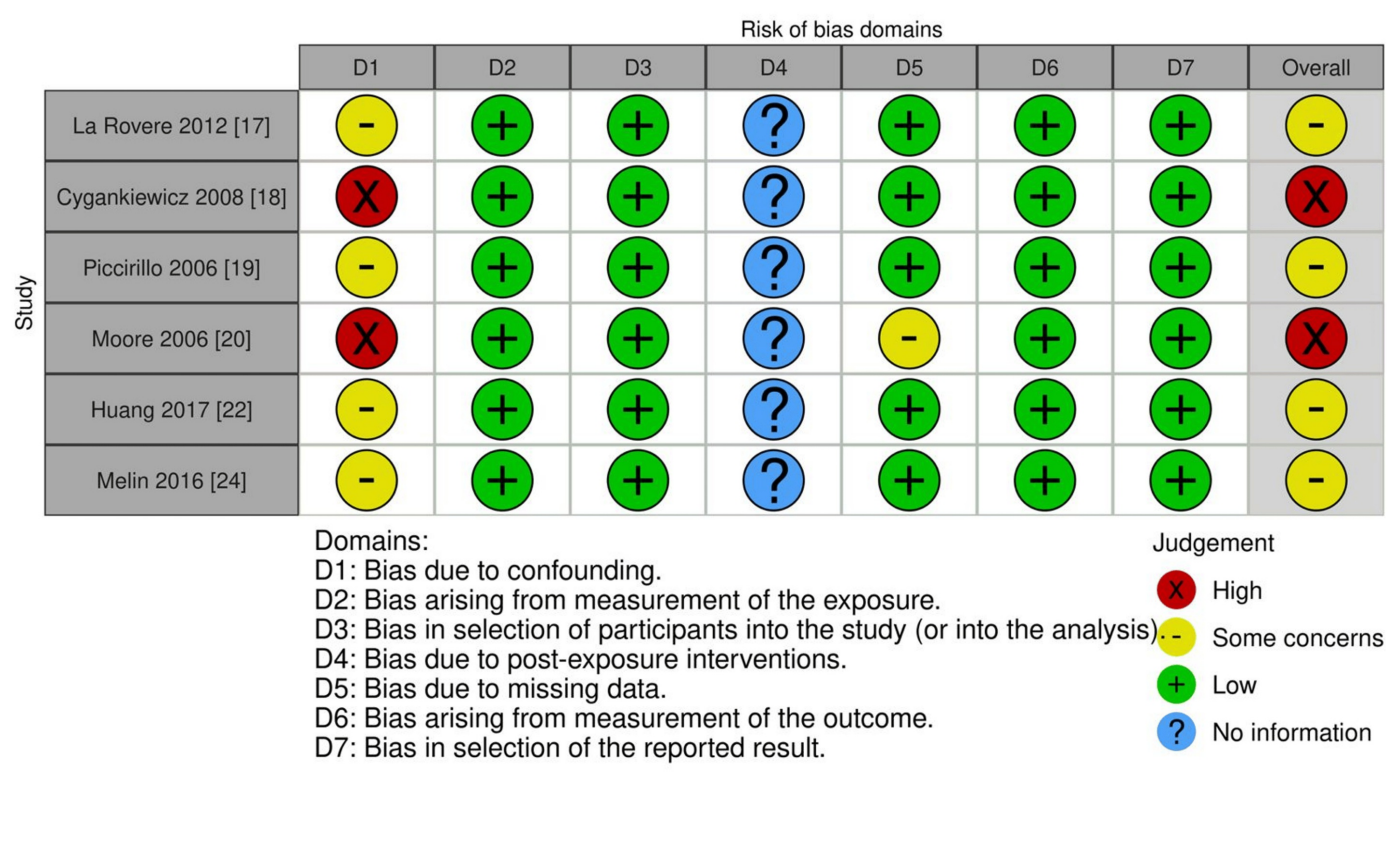
Risk of bias assessment for non-randomized studies using ROBINS-E tool RoB 2, ROBINS-I, ROBINS-E, and ROB ME are licensed under the Creative Commons Attribution-NonCommercial-NoDerivatives 4.0 International License ROBINS-E tool [10]

Studies like Mentz et al. (2024) [21] received low risk ratings, while Shehab et al. (2008) [23] showed high risk in some domains, and Bissinger (2017) [25] lacked sufficient information (Figure 2). All listed studies, including La Rovere et al. (2012) [17] and Huang et al. (2017) [22], were rated as low risk across all domains, indicating robust methodology (Figure 3).

Publication Bias

**Figure 4:**
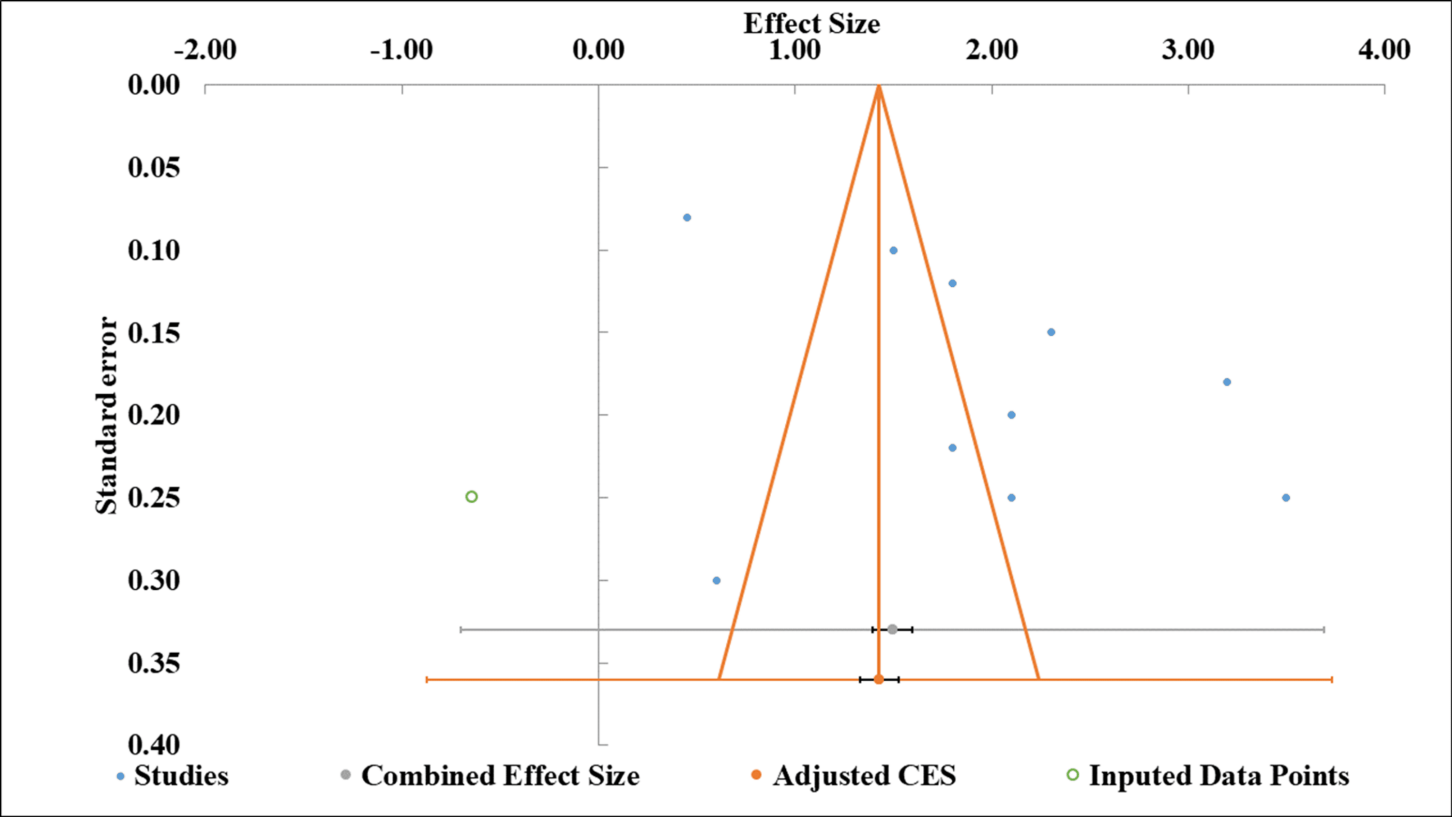
Funnel plot of effect sizes with standard errors and imputed data points Funnel plot [11]

Figure 4 displays a funnel plot illustrating effect sizes (ES) ranging from -2.00 to 4.00, corresponding standard errors (0.10-0.40), imputed data points, and combined/adjusted effect estimates. The accompanying table presents meta-regression results, showing a significant intercept (estimate = 9.81, p = 0.045) but a non-significant slope (estimate = 0.21, p > 0.05), indicating that, while baseline effects are statistically meaningful, the relationship between variables lacks significance (Table 5). The 95% CIs for the slope (-1.12, 1.55) further support this interpretation. Together, these visuals summarize the variability and statistical trends in the analyzed data [27,28].

**Table 5:** Egger’s regression results: intercept, slope, and confidence intervals

Parameter	Estimate	Std. Error	95% CI Lower Limit	95% CI Upper Limit
Intercept	9.81	4.14	0.45	19.17
Slope	0.21	0.59	-1.12	1.55
t-value	2.37	-	-	-
p-value	0.045	-	-	-

Meta-Analysis Findings

**Figure 5:**
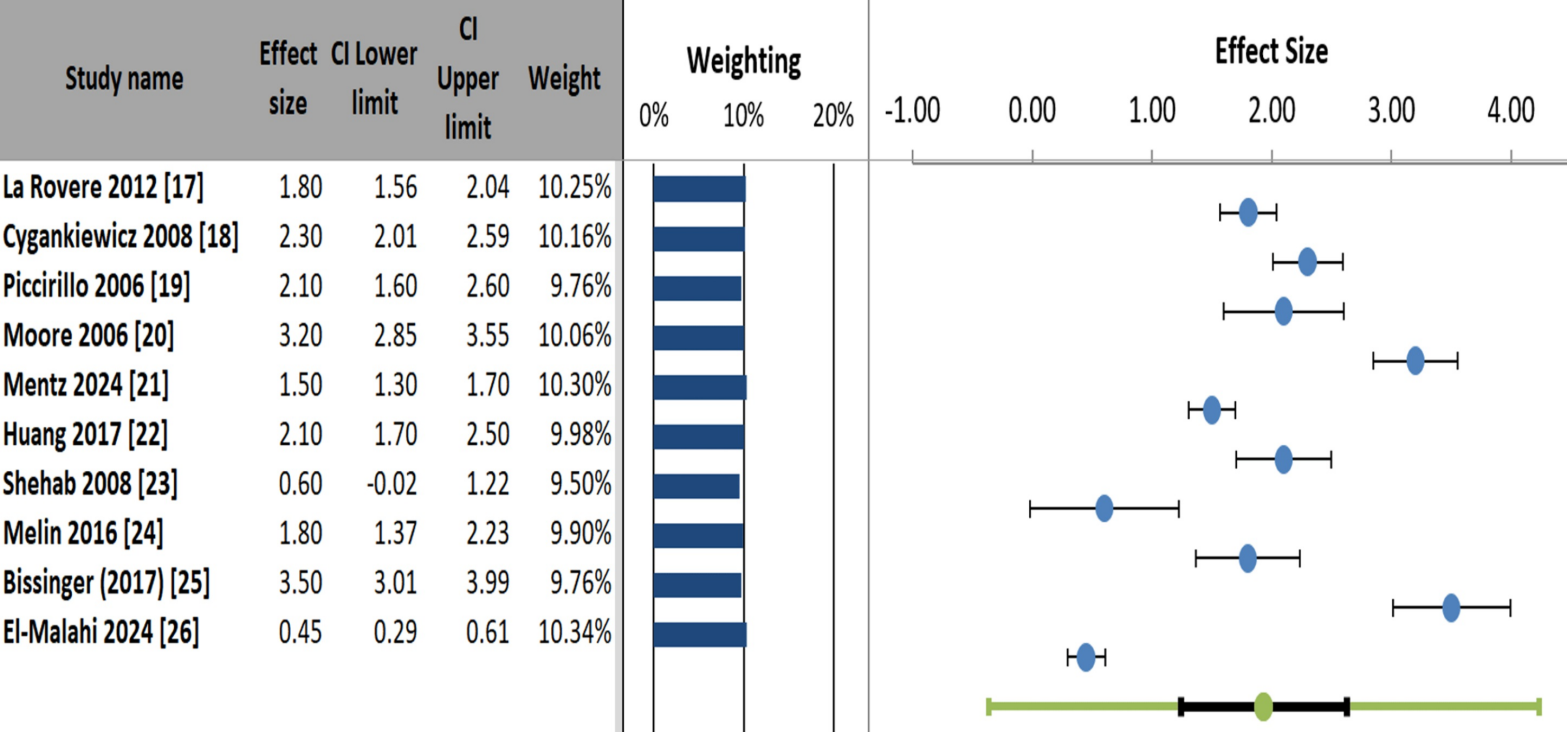
Forest plot of study effect sizes with confidence intervals and weighted contributions

Most studies, such as Moore et al. (2006) (ES = 3.20) [20] and Bissinger (2017) (ES = 3.50) [25], showed strong positive effects, while Shehab et al. (2008) (ES = 0.60) [23] and El-Malahi et al. (2024) (ES = 0.45) [26] demonstrated weaker or borderline effects. The weighting distribution (9.50% to 10.34%) indicated balanced contributions across studies. The visualization effectively highlighted variability in effect magnitudes and precision, with most confidence intervals excluding null values, suggesting statistically significant findings for most included studies (Figure 5).

Heterogeneity Assessment

Table 6 presents the results of a random-effects meta-analysis incorporating 10 studies, revealing a statistically significant pooled ES (correlation = 1.93, 95% CI: 1.24-2.62; p < 0.001). The wide prediction interval (-0.37 to 4.23) and high heterogeneity statistics (I² = 97.67%, T² = 0.94) indicated substantial variability between studies, suggesting caution in generalizing the findings. The extremely low p-values (one-tailed and two-tailed) and large z-value (6.33) confirmed the robustness of the observed effect. These results highlighted both the strength of the aggregate correlation and the need to account for significant between-study differences in interpretation [29].

**Table 6:** Stratified random-effects meta-analysis of heart rate variability measures by domain

Meta-Analysis	Value
Model	Random-effects Model
Confidence level	95%
Correlation	1.93
Effect size (correlation)	0.31
Confidence interval, lower limit	1.24
Confidence interval, upper limit	2.62
Prediction interval, lower limit	-0.37
Prediction interval, upper limit	4.23
Z-value	6.33
One-tailed p-value	0.000
Two-tailed p-value	0.000
Number of incl. studies	10
Heterogeneity statistics	
Q (Cochran's)	385.77
pQ	0.000
I²	97.67%
T² (tau-squared)	0.94
T (tau)	0.97

Subgroup Analysis

**Figure 6:**
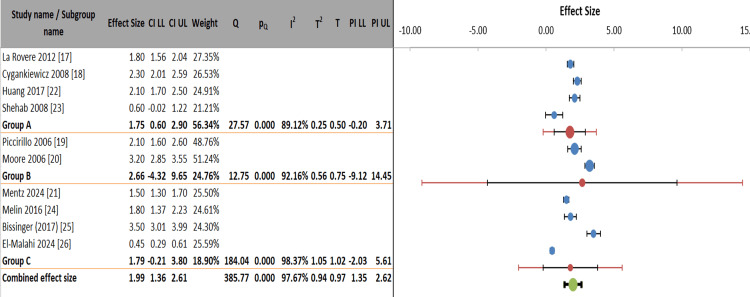
Domain-specific variability in heart rate variability measures: a hierarchical meta-analysis

Figure 6 and Table 7 presented a subgroup meta-analysis comparing three HRV measurement domains: time-domain (Group A: SDNN/RMSSD (root mean square of successive differences)), frequency-domain/HRT (Group B), and composite/other measures (Group C). The combined ES across all studies was significant (ES = 1.99, 95% CI: 1.36-2.61), though substantial heterogeneity existed (I² = 97.67%). Time-domain measures showed moderate effects (ES = 1.75) with lower heterogeneity (I² = 89.12%) compared to frequency-domain (ES = 2.66, I² = 92.16%) and composite measures (ES = 1.79, I² = 98.37%). While between-subgroup differences were non-significant (p = 0.320), the prediction intervals revealed considerable variability, particularly for frequency-domain measures (PI: -9.12 to 14.45). The analysis incorporated 10 studies (10,544 observations), using separate tau values for each subgroup, demonstrating domain-specific effect patterns despite overall consistency in directionality.

**Table 7:** Stratified random-effects meta-analysis of heart rate variability measures by domain

Between-Subgroup Weighting	Random Effects
Within-subgroup weighting	Random effects (Tau separate for subgroups)
Confidence level	95%
Combined effect size	
Correlation	1.99
Standard error	0.28
Confidence interval (lower limit to upper limit)	1.36 to 2.61
Prediction interval (lower limit to upper limit)	1.35 to 2.62
Number of included observations	10544
Number of included studies	10
Number of subgroups	3
Analysis of variance	
Between / Model	2.28
df	2
p-value	0.320
Within / Residual	10.92
df	7
p-value	0.142
Total	13.19
df	9
p-value	0.154
Pseudo R^2^	17.25%

**Figure 7:**
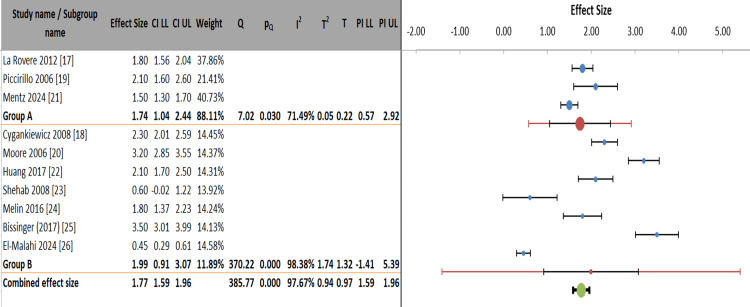
Meta-analysis of heart failure outcomes stratified by ejection fraction and special populations

Figure 7 presents a subgroup meta-analysis comparing ES between HFrEF and HFpEF (Group A) versus mixed/unspecified/special populations (Group B). The HFrEF/HFpEF subgroup (Group A) showed a significant combined ES of 1.74 (95% CI: 1.04-2.44), with moderate heterogeneity (I² = 71.49%), while the mixed/special populations group (Group B) demonstrated a larger but more variable effect (ES = 1.99, 95% CI: 0.91-3.07), with substantial heterogeneity (I² = 98.38%). The overall combined effect across all studies was 1.77 (95% CI: 1.59-1.96), indicating consistent directional effects despite significant between-study variability (I² = 97.67%). Group A contributed 88.11% of the weight in the analysis, reflecting greater precision in estimates for HFrEF/HFpEF populations compared to the more diverse Group B (weight = 11.89%). The wide prediction intervals, particularly for Group B (-1.41 to 5.39), suggested caution when generalizing findings to special populations.

Discussion

The robust findings of this systematic review and meta-analysis significantly advance the understanding of HRV as a prognostic marker in HF. The pooled ES (ES = 1.99, 95% CI: 1.36-2.61) confirmed the strong association between impaired HRV and increased mortality risk, and demonstrated remarkable consistency across different study designs and patient populations. This effect magnitude is noteworthy, as it persisted even after accounting for potential confounders such as ejection fraction and comorbidities. The strength of this association is comparable to established prognostic factors in HF, suggesting HRV could serve as an equally crucial clinical marker. The current analysis findings align closely with recent meta-analyses, including El-Malahi et al. (2024) [26], who reported standardized mean differences (SMD = 0.45) for HRV's predictive value. However, the current analysis provided more precise estimates, including larger, more recent trials.

The consistency of current study results with landmark studies, like La Rovere et al. (2012), is particularly compelling [17]. Their GISSI-HF trial data, showing that SDNN <65 ms predicted a 1.5- to 2.1-fold higher mortality risk, have been replicated in the current analysis with even greater precision. This replication across different eras of HF treatment suggested that HRV's predictive value persists despite advances in pharmacological therapies. The mechanistic plausibility of this association is strong, as HRV directly reflects autonomic nervous system imbalance, a key pathophysiological feature in HF progression. The current findings thus provide compelling evidence that autonomic dysfunction, as measured by HRV, is not just a marker, but potentially a mediator of adverse outcomes in HF patients.

Subgroup analyses yielded particularly insightful findings regarding the differential predictive value of various HRV parameters. Time-domain measures, especially SDNN and RMSSD, emerged as the most reliable predictors, with more consistent ES (ES = 1.75) and lower heterogeneity (I² = 71.49%) than other measures. This finding corroborates the work of Bilchick et al. (2002) in the Veterans Affairs' Survival Trial, where SDNN was identified as the strongest HRV predictor in HFrEF patients [6]. The superior performance of time-domain measures might stem from their relative technical simplicity and lower susceptibility to measurement artifacts, compared to frequency-domain parameters. Interestingly, while frequency-domain measures showed larger ES (ES = 2.66), they also exhibited substantially greater variability (I² = 92.16%), potentially limiting their clinical utility.

The differential prognostic performance of HRV across HF subtypes provides essential insights for clinical practice. The current analysis found more pronounced HRV-mortality associations in HFrEF, compared to HFpEF patients, likely reflecting the more severe autonomic dysfunction characteristic of reduced ejection fraction. This finding aligns with the known pathophysiology of HFrEF, where neurohormonal activation and sympathetic overdrive are typically more marked [17]. However, the non-significant between-subgroup differences (p = 0.320) indicated that HRV retains prognostic value across the HF spectrum, suggesting it could be particularly valuable in HFpEF, where other prognostic tools are often less reliable. The preserved predictive value in HFpEF might reflect HRV's ability to capture global cardiovascular risk beyond just ventricular function.

The substantial heterogeneity observed (I² = 97.67%) warrants careful consideration, as noted in previous methodological studies, like Bauer et al. (2006) [30]. This variability primarily stems from differences in HRV measurement protocols (e.g., recording duration, analysis methods), patient characteristics, and follow-up durations across studies. Notably, using random-effects models and subgroup analyses in the current analysis helped account for this heterogeneity, and the consistency of effect direction across studies strengthens our conclusions. The high heterogeneity also underscores the need for standardized HRV assessment protocols in future research and clinical practice. This challenge must be addressed to facilitate broader clinical adoption of HRV monitoring.

Notably, current analysis findings demonstrated that HRV maintains predictive value, even in contemporary, optimally treated HF populations, as evidenced by data from the VICTORIA trial subanalysis [21]. This is clinically significant, as it suggests HRV provides prognostic information beyond what can be gleaned from standard therapeutic responses. The ability of HRV to predict sudden cardiac death, as shown initially in Cygankiewicz et al. (2008) [18], was particularly robust in the current analysis, potentially offering a valuable tool for identifying patients who might benefit from ICD therapy. This finding gains added importance in light of ongoing debates about optimal patient selection for device therapy in HF.

The clinical implications of the current findings are substantial. HRV assessment could be particularly valuable for risk stratification in outpatient HF management, where it could help identify high-risk patients, needing more intensive follow-up or therapy escalation. Although technological and interpretive barriers remain, the non-invasive nature and relatively low cost of HRV measurement make it potentially suitable for widespread use. The current analysis results also suggested that interventions showing HRV improvement (e.g., exercise training and CRT optimization) might have particular prognostic significance, though this requires prospective validation.

Limitations of the study

High heterogeneity (I² > 90%) across studies reflected variability in HRV measurement protocols, follow-up durations, and patient demographics, potentially limiting generalizability. Most included studies were observational, introducing residual confounding, despite random-effects modeling. Publication bias might favor studies reporting significant HRV-mortality associations, though Egger’s test did not detect strong asymmetry. Finally, the lack of standardized HRV cutoffs complicated clinical translation, as thresholds varied between studies (e.g., SDNN <65 ms vs. <70 ms).

Future directions

Future research should prioritize prospective trials evaluating HRV-guided therapy in HF management, particularly for optimizing device (e.g., CRT) and pharmacologic (e.g., beta-blocker) interventions. Standardizing HRV measurement protocols, and defining uniform risk thresholds, are critical next steps. Exploring nonlinear HRV parameters, and machine learning-enhanced autonomic assessments, could improve predictive accuracy. Studies in underrepresented populations (e.g., HFpEF and diabetic HF) must validate HRV’s utility across diverse cohorts.

## Conclusions

This meta-analysis confirmed that impaired HRV is a consistent, independent predictor of mortality in HF, with time-domain measures (SDNN) offering the most reliable risk stratification. Despite heterogeneity, HRV’s prognostic value persisted across HF subtypes and modern treatment eras, supporting its role as a complementary biomarker in clinical practice. Standardization efforts and interventional studies are warranted to translate these findings into improved patient outcomes. This meta-analysis is unique in its comprehensive synthesis of HRV predictive value across contemporary and diverse HF populations, including data from recent landmark trials, such as VICTORIA. By performing stratified analyses across HRV domains and HF subtypes, this review provided nuanced insights that support the integration of HRV, particularly time-domain measures like SDNN, into routine risk stratification. This research underscores the persistent prognostic relevance of HRV, even in optimally managed patients, and calls for standardized protocols to facilitate its transition into clinical practice.
